# Multivariate statistics and hydrogeochemistry of deep groundwater at southwestern part of Bangladesh

**DOI:** 10.1016/j.heliyon.2022.e11206

**Published:** 2022-10-22

**Authors:** Tusar Kumar Das, Shakir Ahmed, Alamin Hossen, Md. Hasibur Rahaman, Mohammad Mahfuzur Rahman

**Affiliations:** a Department of Environmental Science and Technology, Jashore University of Science and Technology, Jashore, 7408, Bangladesh; b International Program in Hazardous Substance and Environmental Management, Chulalongkorn University, Bangkok 10330, Thailand

**Keywords:** Groundwater, Hydrogeochemical classification, Spatial distribution, Principal component analysis (PCA)

## Abstract

Multivariate statistics and GIS alone with geochemical modeling were applied to investigate the hydro-geochemical characteristics of groundwater and their spatial distribution in the deep aquifer system of Bagherpara Upazila, Bangladesh. This Upazila consists of an area of 308.3 km^2^ and local people mostly rely on groundwater to meet the drinking water requirements. Water samples from one hundred randomly selected deep tube wells (152–198 m) were collected and analyzed for 14 water quality parameters to characterize the hydro-geochemical properties. The groundwater shows slight alkaline in nature throughout the study area. Most of the water samples were turbid and 68% of them failed to meet the drinking water quality standard prescribed by the World Health Organization (WHO). TDS concentration ranges between 280 mg/L and 1040 mg/L, with a mean value of 446.20 (±122.19) mg/L. The general order of cation and anion along the study area were Ca^2+^>Mg^2+^>Na^+^>K^+^ and HCO_3_^-^>Cl^−^>PO_4_^3-^>SO_4_^2-^>NO_3_^-^ respectively. Carbonate weathering is the dominant process for releasing ions in groundwater. Besides, the ion exchange process is active in the study area, which leads to the reduction of Na^+^ ions. Gibb's plot suggests a rock dominance inheritance controls the dissolution and precipitation of minerals along with the ion-exchange process, and ultimately dictates the groundwater chemistry. Besides, the Piper diagram showed that Ca^2+^-Mg^2+^-SO_4_^2-^ is the dominant water type in 65% of the samples followed by Ca^2+^-Mg^2+^–HCO_3_^-^ water type (35%). The abundance of Ca^2+^ and Mg^2+^ ions and the alkaline nature of groundwater indicate mixed geochemical facies and reverse exchange reactions. The principal component analysis (PCA) reveals that weathering and leaching of host rocks was the main natural source, while municipal solid waste dumping, sewage discharge, and fertilizer application could be other anthropogenic factors that affect the groundwater geochemistry. In fine, the chemical characteristics of groundwater were acquired through rock dissolution, percolation, and reverse exchange process.

## 1 Introduction

The quality of water is essential for human well-being. This water is usually acquired from two vital sources, groundwater and surface water (Boateng et al., 2016). Irrespective of sources, we should consider the suitability or potability of water before usage. Most nations have their water quality standards, specifying the maximum allowable limits on the physical, chemical, and biological parameters depending on the use. Besides, several countries also adopted the standards proposed by the World Health Organization (WHO) and the United States Environmental Protection Agency (USEPA) (Akter et al., 2016). Water is often evaluated as risky for human health once any of its parameters exceeds acceptable limits (WHO, 2012). However, the contaminated water can not only be a threat to human health but also to ecosystem functioning, maintaining biological integrity, and the capability to render ecosystem services (Howladar et al., 2018). Considering its multiple criteria and diversity of usage, groundwater is recognized as the safest source of water.

At present, the increasing demand for drinking, industrial, agricultural and domestic water is mostly met from groundwater resources (Gupta et al., 2009). But ensuring the use of groundwater for those purposes, its quality must be determined. The groundwater quality of a particular region is a function of chemical and physical parameters. The geological formations and anthropogenic activities greatly influence variation in groundwater quality (Gupta et al., 2009; Selvakumar et al., 2017). Another important fact is that groundwater doesn't exist in a single widespread aquifer beneath the earth's surface, rather occurs as a continuum of several small-scale aquifers with similar properties. The quality of water of a specific aquifer greatly depends on the recharged water quality, surface water, precipitation, and sub-surface geochemical processes. Additionally, hydrologic and human factors can cause a periodic change in groundwater quality (Vasanthavigar et al., 2010). During the movement of groundwater through the rocks' pore spaces, a significant chemical reaction occurs that exists in the geo-chemistry of underground water (Amadi et al., 2012).

Geochemistry uses the principles of chemistry for explaining the mechanisms that occur in geological systems such as the Earth's crust (Fowler, 2004). The geochemistry of groundwater is determined by different factors such as geology, quality of input water from different sources, and chemical weathering of rocks. Those processes are the responsible factors for changes in groundwater quality and chemistry (Subba and Surya, 2010; Mostafa et al., 2017). The geochemical process in groundwater is identified by the major ion chemistry of groundwater, which is controlled by weathering of minerals within the rocks (Jacks, 1973; Bartarya, 1993). Therefore, the dissolution of undesirable minerals in the waters is not controllable once they enter the ground. The deficiency or excessive intake of those major, minor, and trace elements in drinking water can have a significant impact on public health (Frengstad et al., 2001). The effects of contaminated groundwater on public health are frequently observed in different parts of the Bengal Basin.

The Bengal Basin (West Bengal and Bangladesh) is a habitat of about 256 million populations. About 85% populations of this densely populated sedimentary basin use groundwater for drinking, irrigation, and household purposes (Bahar and Reza, 2010; Worland et al., 2015; Haque et al., 2015). The aquifer system of this region is developed from the deposits of unconsolidated shallow Pleistocene to Recent fluvial and estuarine sediment. Bangladesh's part of this alluvial aquifer system meets 99% of the drinking water and 90% of the irrigation water demand (Zahid et al., 2008). The southwestern part of Bangladesh is usually plain and formed from the Holocene sedimentation of the mighty Ganges River system, which characterized the geology of this area. Both Pleistocene older alluvium and Holocene sediments are two types of geological structures that exist along the area. The soil is generally composed of clay, fine sand, and mud, while the color is gray and brown (Dowling et al., 2002; Haque et al., 2015). The surface geology of this region is categorized as a Ganges flood plain, but an active Ganges flood plain is still observed in the outer shell area. The newly developed flood plain is composed of fine-medium grain sand, silt, and clay and is relatively loose and fragile (Islam et al., 2015). The sea-level change in the Holocene period controlled the sedimentation in the plain and leads the formation of several surface features (Ravenscroft et al., 2005). The most important fact is that the groundwater quality of this sedimentary basin is characterized by late Quaternary stratigraphy, geomorphology, and sedimentation (Hasan et al., 2007; Muley et al., 2010).

Therefore, the objective of this research is to characterize groundwater chemistry and correlate between hydrochemistry of the groundwater and aquifer geology of the southwest part of the Bengal delta using multivariate statistical methods. This method is a useful analytical technique for the interpretation of water quality data from large environmental datasets and helps to identify the important natural and anthropogenic processes controlling groundwater quality (Helena et al., 2000; Islam et al., 2019). For example, principal components analysis (PCA) is one of the widely used multivariate statistical methods, that helps to categorize specific hydro chemical facies and dominant hydro chemical processes. Finally, PCA, Scatter diagram, Gibbs plot, and Piper diagram are used to identify the controlling factor of groundwater geochemistry (Kumar et al., 2011; Ghesquière et al., 2015).

## 2 Materials and methods

### 2.1 Study area

**Figure 1:**
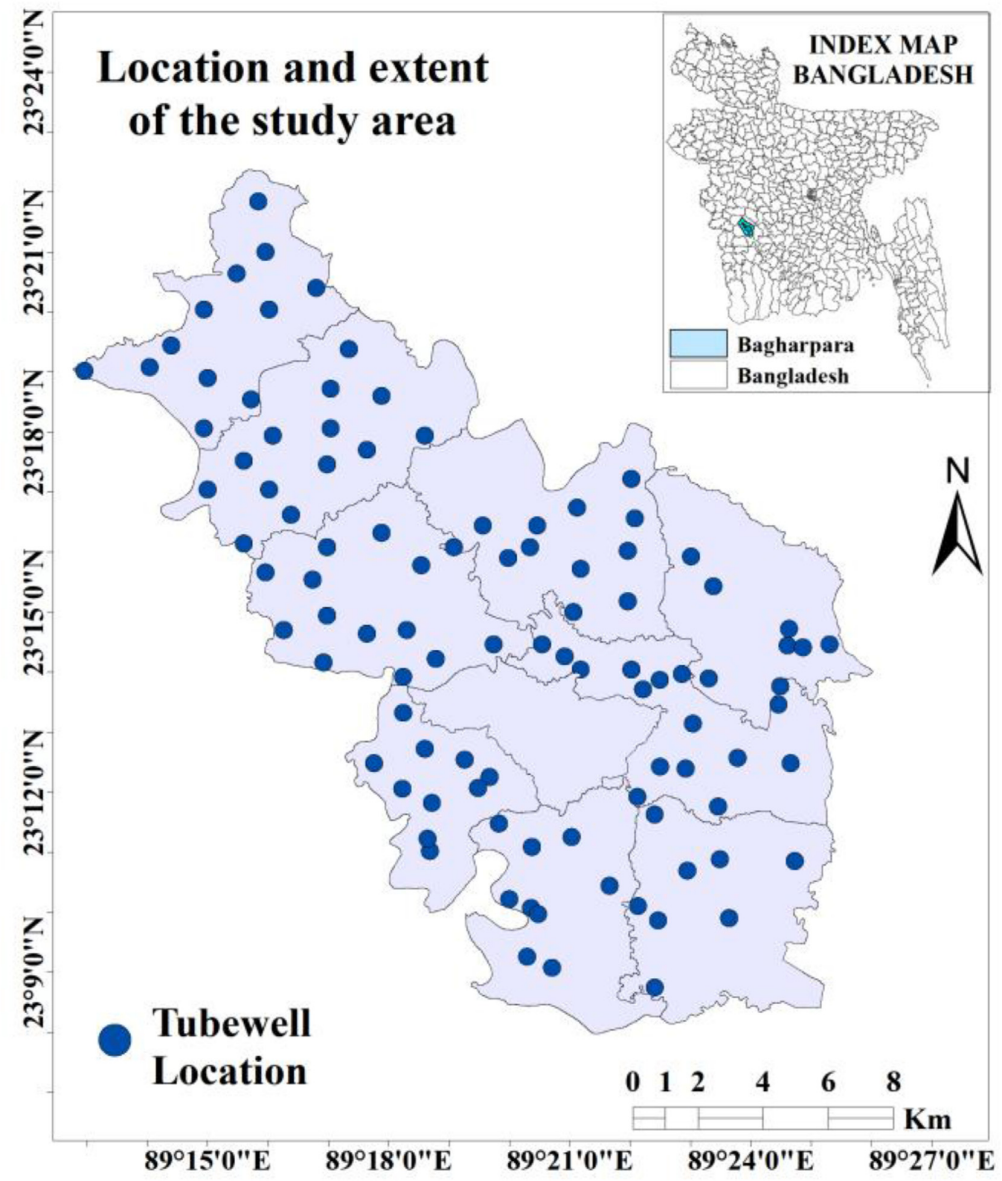
Location map of the study area and location of sampling point (Tube well).

The study area is situated in the southwestern part of Bangladesh. The study area is located between 23°08′ to 23°21′ N and 89°13′ to 89°26′ E, with an area of 308.3 km^2^ (Figure 1). The total population of this area is 216,897. This area is situated in a humid tropical region and is characterized by six different seasons. The annual temperature in this region ranges from 15.4 to 34.6 °C and the average annual precipitation is 1537 mm, which is mainly concentrated from April through October (78% of total annual precipitation) (Hassan et al., 2020). The humidity ranges from 68.6% to 90% in various seasons of the year. November through February is usually dry winter and receives a little fraction of total precipitation (Tofayal et al., 2020).

The Bhairab is the main river system of the study area, which is a tributary of the Ganges River system. Geologically this area is termed as the Ganges River floodplain with a plain topography (Brammer et al., 1988). Two distinct geological formations Holocene sediments and Pleistocene older alluvium exist in this region. Generally, the plain land of the study area is formed by the Holocene floodplain. The shell area of this region is loose and fragile and categorized as an active flood plain (Haque et al., 2015; Islam et al., 2015; Huq et al., 2018). The alluvium soil of this region is mainly composed of silty clay, silt, and fine sand, which is found from the surface to 300 m depth (Bahar and Reza, 2010). Considering diverse soil characteristics of soil three distinct aquifer system is identified in the study area such as upper (50–100 m from the surface), main (250–350 m from the surface), and deep aquifers (below 250 m) (Datta and Ghosh, 2015). The predicted storage coefficient, transmissivity, and specific yield of this region are 2 × 103, 3900 m^2^/day, and 16.6% respectively (Bahar and Reza, 2010; Ahmed et al., 2020).

### 2.2 Water sample collection and analysis

The deep tube-well (depth 152–198 m) water samples were collected from 100 randomly selected sampling stations in December 2019. Potable water bottles (500 mL) were used in sample collection. The bottles were socked in 0.1 N HNO_3_ after being prewashed with detergent and tap water. After that, bottles were washed with distilled water and oven-dried at 60 °C. To avoid probable contamination during sampling, the bottle was rinsed properly with sample water. Before sample collection, the tube well was pumped to exact times concerning for the depth in feet (i.e. 600 pumps for a 600 feet tube well). After collection, the samples were instantly carried Environmental Chemistry Laboratory of Jashore University of Science and Technology and analyzed. Some parameters like temperature, pH, TDS, and EC were analyzed at the sampling site by using a microprocessor pH meter (model- HANNA instrument pH 211) and conductivity meter (model- HI 8033). The concentration of Na^+^ and K^+^ were analyzed by using Flame Photometer (flame photometer- PEP7). The Ca^2+^, Mg^2+^, and HCO_3_^-^ concentrations were measured by titration colorimetric method and Cl^−^ by an ergonometric method. The NO_3_^-^, PO_4_^-^, and SO_4_^2-^ were measured by Spectrophotometer (model- UV-visible spectrophotometer, helios 949923045811) (APHA, 1995).

### 2.3 Statistical and hydrogeochemical analysis

All statistical analysis were performed by using SPSS version 20. ArcGIS version 10.5 was used to prepare a location map of the sampling point and spatial distribution of ions along the study area. An inverse distance weighted (IDW) algorithm was used to interpolate data in a surface map (Singh et al., 2011). Principal Component Analysis (PCA) is a dimension reduction method that converts raw data into a form that can be evaluated in a multidimensional space (Jolliffe, 2002). This method transforms a highly correlated large number of variables into minor uncorrelated variables, *i.e.*, principal components (PCs) (Kouras et al., 2007). The extracted principal components show original form with a minimum loss of information (Singh and Singh, 2018). The PCA is likewise a beneficial tool in offering concepts of a sub-surface geochemical system (Singh *et al.*, 2017). The relationship between water composition and aquifer lithological characteristics is mostly represented by using Gibbs Diagram. Gibbs ratio I (for anion) = Cl^−^/(Cl^−^ + HCO_3_); Gibbs ratio II (for cation) = Na^+^/(Na^+^+Ca^+2^), where all the ionic concentration were expressed in meq (Gibbs et al., 1970).

## 3 Result and discussion

### 3.1 Hydro geochemistry of groundwater along the study area

**Figure 2:**
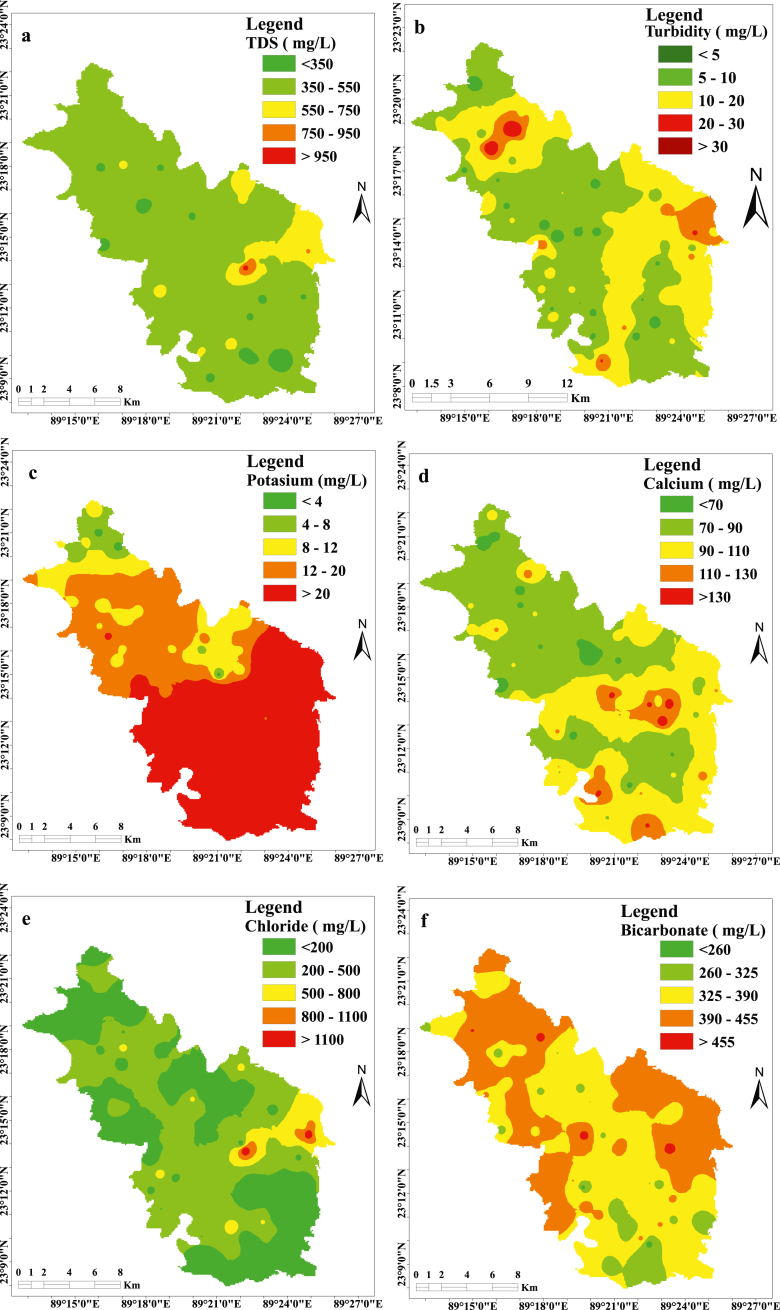
Spatial distribution map of **(a)** TDS, **(b)** Turbidity, **(c)** K^+^, (d) Ca^2+^, **(e)** Cl^−^ and **(f)** HCO_3_^−^in the study area.

The analyzed physicochemical parameters of underground water were statistically summarized and compared with WHO and the Department of Environment (DoE) drinking water quality standards in Table 1. The pH value ranges from 7.0 to 7.7 with an average value of 7.25, which indicates the slight alkaline dominance in the investigated sample. The pH value in the groundwater of the Jashore region generally ranges from 6.80 to 8.0 (Hassan et al., 2020) but in the sedimentary basin of southwestern Bangladesh, it ranges from 6.0 to 8.20 (Datta and Ghosh, 2015; Das et al., 2017; Das *et al.*, 2021b). This was due to the abundance of calcic plagioclase, micas, carbonates, clay, and quartz minerals (Datta and Ghosh, 2015). The turbidity of the water sample ranges from 0.34 to 49.4 NTU with a mean value of 9.82 (±8.49) NTU, where 68% of the sample exceeds the WHO recommended value of turbidity for drinking water. The turbidity shows an almost homogeneous distribution in the study area with little elevated value in the southern part (Figure 2b). The EC and TDS indicate the salinity of water, where nonionic constituents are absent in water (Rahman et al., 2018). The measured EC value range from 570 to 2100 μS cm^−1^ with a corresponding average of 891.80 (±242.45) μS cm^−1^, while 73% of the sample exceeded the prescribed value of WHO. Groundwater EC shows a large-scale regional trend in Southwestern Bangladesh. EC value showed an increasing tendency from north to south in coastal Bangladesh (Naus et al., 2019). The concentration of TDS ranges from 280 to 1040 mg/L, with an average value of 446.20 (±122.19) mg/L, where 18% of the water samples exceed the WHO desirable limit. Due to elevated concentrations of TDS (TDS> 1000 mg/L), most water samples can be considered as brackish water types (Freeze and Cherry, 1979). The spatial distribution of TDS evident no significant variation in the study area (Figure 2a). This represents almost spatial homogeneous aquifer characteristics (Zahid et al., 2018).

**Table 1:** Chemical composition of groundwater sample along the study area.

Variable	Min.	Max.	Mean (±SD)	Standards		% above WHO limits
				WHO	DoE	
pH	7.00	7.7	7.25 (±0.14)	6.5–8.5	6.5–8.5	0
Turbidity (NTU)	0.34	49.40	9.82 (±8.49)	5	10	68
EC (μS cm^−1^)	570	2100	891.80 (±242.45)	750	300–1500	73
TDS (mg L^−1^)	280	1040	446.20 (±122.19)	500	1000	18
Na^+^ (mg L^−1^)	9.26	48.17	21.28 (±7.84)	200	200	0
K^+^ (mg L^−1^)	2.90	92.73	31.13 (±23.08)	30	12	44
Ca^2+^ (mg L^−1^)	52.00	144.00	89.10 (±19.42)	75	75	24
Mg^2+^ (mg L^−1^)	14.40	81.60	43.78 (±16.54)	30	30–35	73
PO_4_^3-^ (mg L^−1^)	0.22	62.36	20.04 (±22.71)	−	6	−
SO_4_^2-^ (mg L^−1^)	0.28	9.35	4.62 (±4.61)	250	400	0
NO_3_^-^ (mg L^−1^)	0.25	8.83	1.48 (±1.07)	50	10	0
HCO_3_^-^ (mg L^−1^)	237.90	494.10	379.79 (±49.85)	100	−	100
Cl^−^ (mg L^−1^)	17.73	1471.18	264.10 (±255.83)	250	150–600	29

The concentration of the analyzed cation showed the order of Ca^2+^>Mg^2+^>Na^+^>K^+^, where the relative contribution is 45.22%, 37.03%, 9.66%, and 8.10% respectively. A similar pattern was found in the northern part of the Jashore district (Hassan et al., 2020). The minimum and maximum concentrations of Ca^2+^ are 52.0 and 144.0 mg/L respectively, while the average concentration was 89.10 (±19.42) mg/L. About 24% of the sample exceed the acceptable limit (75 mg/L) prescribed by WHO and DoE. In most of the sampling stations, the Ca^2+^ concentration was higher than Mg^2+^ indicating the dominance of calcium-bearing minerals in the sedimentary basins like limestone, dolomite, calcite, feldspar, etc (Yadav et al., 2018). The gradual increase of Ca^2+^ concentration from north to south along the study area is observed in spatial distribution (Figure 2d). The value of Mg^2+^ varies from 14.40 to 81.6 mg/L, where the average concentration was 43.78 (±16.54) mg/L. The WHO prescribed limit of Mg^2+^ in drinking water was 30 mg/L but in the study area 73% of exceeds the limits. The dissolution of Mg^2+^ bearing minerals in rocks was probably responsible for a higher concentration of Mg^2+^ in groundwater. Other probable sources would be industrial wastes, domestic and animal waste (Bodrud-doza et al., 2019).

The Na^+^ content in groundwater varies from 9.26 to 48.17 mg/L, where the mean concentration was 21.28 (±7.84) mg/L and the entire sample were within WHO and DoE prescribed limit. The Na^+^ ion shows conservative nature as it easily binds with clay minerals through an ion exchange process (Subramani and Saxena, 1983). Sodium and potassium concentration in groundwater is proportional to its salinity (El Ghandour et al., 1983). Salinity along the study area is very low (mean TDS 446.20) that's why both sodium and potassium are in limiting conditions compared to other cations. Research shows that in the south-western part of Bangladesh deep aquifer (150–300 m) is formed from Pleistocene sediments, which contains high percentage potassium enriched minerals like shales (26600 ppm), sandstone (10700 ppm) and carbonate (2700 ppm) in some area (El Ghandour et al., 1983; Worland et al., 2015; Islam et al., 2019). Groundwater in the Sahel region of Africa contains 1392 mg/L of potassium, and this area also contains potassium-enriched minerals (Modibo Sidibé et al., 2019). Gibbs diagram shows that mineralization is the only source of sodium in this aquifer, while other factors like erosion of salt deposits, surface water infiltration, saltwater intrusion, irrigation water leaching, and groundwater pollution by sewage effluent are not active in this region. The K^+^ was the fourth dominant cation species in the study area whose concentration is between 2.90 to 92.73 mg/L. The K^+^ shows heterogeneous distribution along the study area (Figure 2c). Its concentration significantly increases from north to south direction and its main source is weathering of potash and silicate minerals (Yadav et al., 2018). About 44% of the sample exceeds the WHO prescribed drinking water quality standard.

The anionic dominance pattern was HCO_3_^-^>Cl^−^>PO_4_^3-^>SO_4_^2-^>NO_3_^-^ and their relative contribution is 53.72%, 43.42%, 1.80%, 0.90% and 0.16% respectively. Another study in the southwestern coastal region shows a similar anionic dominance pattern (Das et al., 2021a). The minimum and maximum concentrations of HCO_3_^-^ were 237.90 and 494.10 mg/L respectively, while the average concentration was 379.79 (±49.85) mg/L. Its concentration gradually decreases from north to south (Figure 2f). Generally, HCO_3_^-^ in underground water represents fresh water but high Cl^−^ concentration is influenced by seawater intrusion (Kumar et al., 2014). The high concentration of Ca^2+^ and HCO_3_^-^ indicate that they originate from the same source of minerals like limestone, chalk, dolomite, etc (Adimalla, 2020). Cl^−^ is the second-largest dominant anion species and its concentration varies from 17.73 to 1471.18 mg/L with an average value of 264.10 (±255.83) mg/L. Generally, Cl^−^ is considered as an index of water quality and its high concentration makes the water salty and has laxative effects. About 29% of water samples exceed the WHO drinking water quality standard for chloride. The most important fact is that Cl^−^ concentration gradually increases from north to south in the study area (Figure 2e). This is the usual distribution pattern of Cl^−^ in the southwestern part of Bangladesh (Ahmed et al., 2020). A diverse natural procedure like weathering, dissolution of deposited salt, and seawater intrusion are liable for high Cl^−^ in groundwater (Bhat et al., 2016). The high evaporation rate and influence of sea-water intrusion were probably responsible for the high Cl^−^ concentration in the study area (Ahmed et al., 2020).

The SO_4_^2-^ is the fourth dominating anion species and its concentration ranges from 0.28 to 9.38. Different studies in the southern part of Bangladesh also found a similar concentration of SO_4_^2-^ in deep underground water (Islam et al., 2017). The SO_4_^2-^ concentrations in groundwater samples were very low, which indicates the absence of sulfate-rich rocks like gypsum in the aquifer. Furthermore, the low sulfate concentration suggests that the area was not industrial where higher levels could be anticipated due to industrial processes and contaminants (Egbueri, 2019). The concentration of NO_3_^-^ and PO_4_^3-^ is negligible along the study area. Those are major plant nutrient that comes from fertilizer application. The occurrence of elevated nitrate concentration in drinking water raises the risk of gastric cancer and other possible health hazards to infants and pregnant women (Todd, 1980). However, our results showed negligible concentrations and no possibility to affect by these types of diseases.

### 3.2 Rock weathering and dissolution

**Figure 3:**
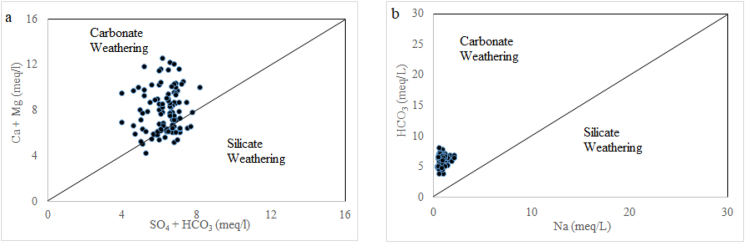
Scattered diagram of selected ions. **(a)** Ca^2+^+Mg^2+^ vs SO_4_^2-^ + HCO_3_^-^ scatter plot **(b)** HCO_3_^-^ vs Na^+^ plot.

This comparison diagram was performed to identify the domination of rock water interaction or identification of ion source along the study area. The most common cations (Mg^2+^, Ca^2+^, and Na^+^) in groundwater generally originate from the weathering and degradation of minerals such as carbonate, silicate, and sulfate-bearing minerals (Hwang et al., 2017; Karunanidhi et al., 2020). The plot Ca^2+^+Mg^2+^ versus SO_4_^2−^+HCO_3_^−^ shows the dominating weathering processes along the study area (Figure 3a). The majority of sampling point in Figure 3a falls above the 1:1 line. This indicates that carbonate weathering is the dominant process for releasing Ca^2+^ and Mg^2+^ ions in groundwater, while few samples that fall below the line suggest silicate weathering. It is suggestive that the weathering of gypsum, calcite and dolomite types of minerals was responsible for releasing those ions into groundwater (Sonkamble et al., 2012). Another important fact is that the point below the 1:1 line signifies that SO_4_^2−^ in groundwater originates from the dissolution of Glauber's salt. This salt dissolution can also contribute to Na^+^ ions in groundwater (Eqs. (1) and (2)) as per suggested by Li et al. (2016).

(1) Na_2_SO_4_.10H_2_O = 2 Na^+^ + SO_4_^2-^ + 10 H_2_O

(2) CaSO_4_.2H_2_O = Ca^+^ + SO_4_^2-^ + 2 H_2_O

The sampling point falling along the 1:1 line indicates both carbonate and sulfate minerals are equally responsible for releasing those ions (Li et al., 2014). But in (Na^+^ Vs HCO_3_^−^) all the samples fall above the 1:1 line (Figure 3b). The dissolution of carbonate-bearing minerals is mainly responsible for releasing Na^+^ ions in groundwater. Weathering of dolomite, calcite, and gypsum in the aquifer was the dominant phenomenon in the study area (Das et al., 2021a). But the important fact is that the elevated concentration of HCO_3_^-^ in groundwater compared to Na^+^ ion implies silicate weathering; the high HCO_3_^-^ level is well-suited for this process (Fisher and Mullican, 1997). The ion exchange process can lead to the reduction of Na^+^ ions in groundwater (Li et al., 2016a).

### 3.3 Gibbs Diagram

**Figure 4:**
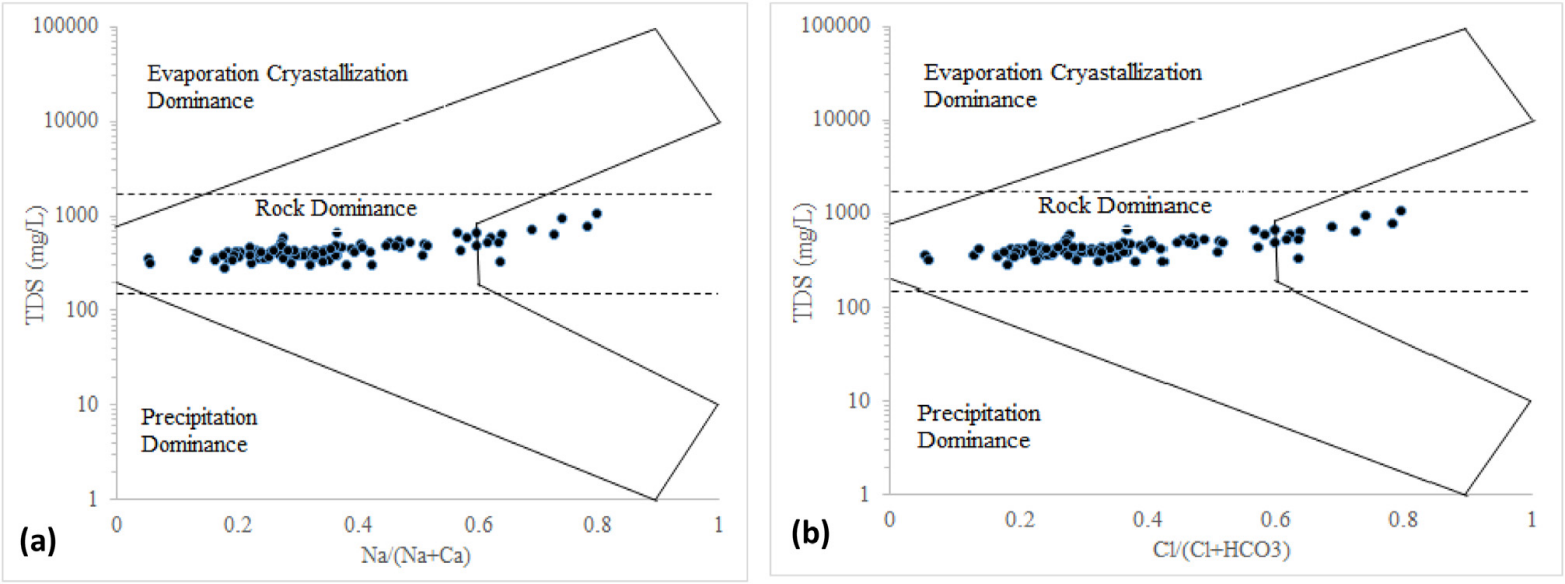
Gibb's plot indicating the predominant geochemical process in underground water samples along the study area. **(a)** TDS versus Na^+^/(Na^+^ + Ca^2+^); **(b)** TDS versus Cl^−^/(Cl^−^ + HCO^3-^).

Gibbs diagrams (Gibbs et al., 1970) are commonly used for underground water analysis across the globe (Adimalla and Wu, 2019). The most important factor is that this plot doesn't depict anthropogenic impacts on hydro-geochemistry. It provides significant information to determine the geochemical factors controlling underground water hydro-geochemistry (Li et al., 2016a, Li et al., 2016b). According to this diagram three distinct process precipitation dominance, rock dominance and evaporation dominance controls hydro-geochemistry (Gibbs et al., 1970; Adimalla et al., 2018). The diagram suggests that rock dominance is the predominant factor in controlling groundwater geochemistry along the study area (Figure 4a & b). Some sample shows an increasing tendency towards evaporation dominance, suggestive of an increasing concentration of chemically weathered ions (Nazzal et al., 2014).

### 3.4 Geochemical facies

**Figure 5:**
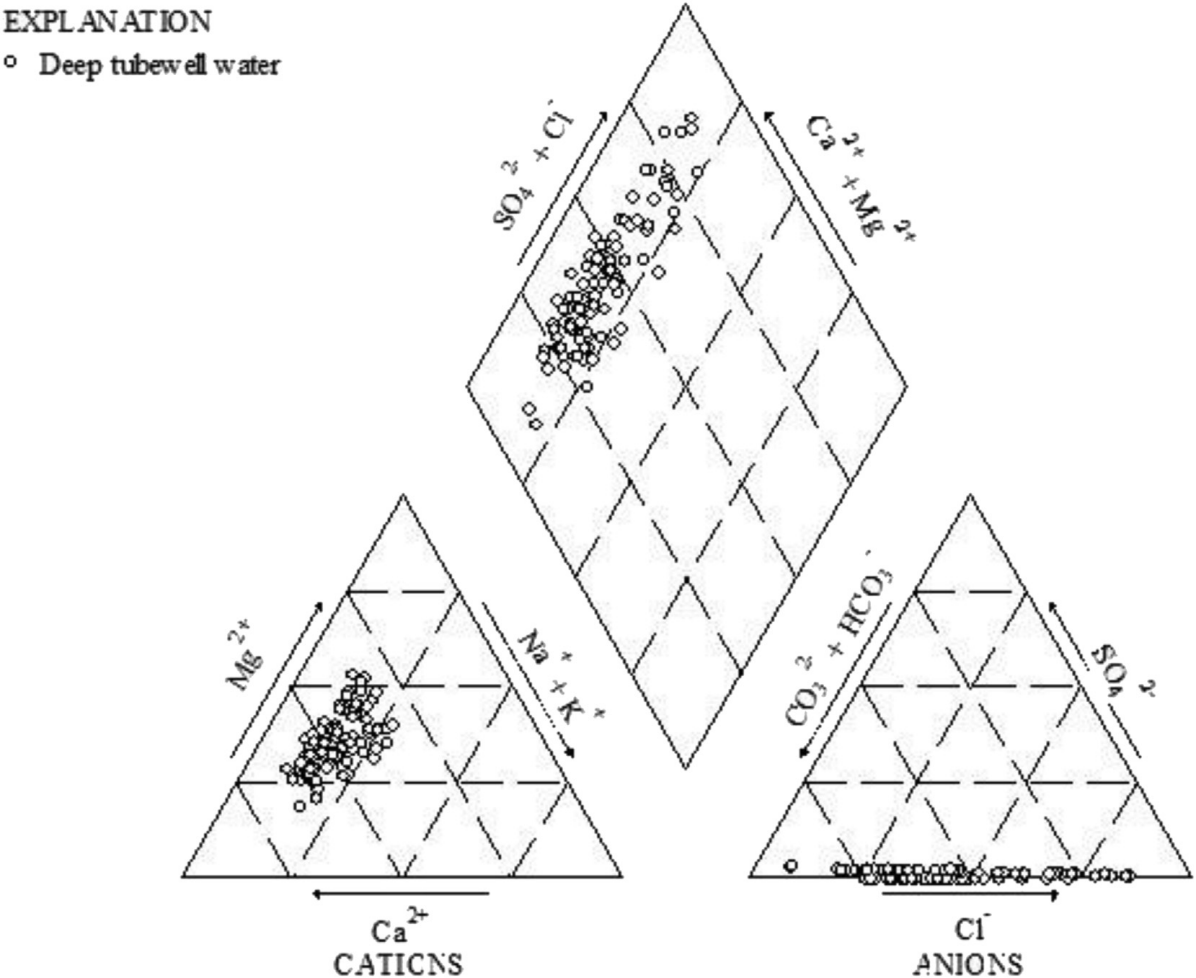
Pipertrilinear plots illustrating the geochemical facies of groundwater chemistry along the study area.

The underground water geochemistry is inferred by using the piper trilinear diagram (Piper, 1944). The concentration of cation and anion was expressed in meq/l and plotted in the piper diagram. It is a very useful tool to identify the evolution of groundwater and its chemical relationship along the study area (Adimalla and Wu, 2019). The underground water is dominated by alkaline earth exceeding alkalis and strong acids exceed weak acids. The piper diagram classifies the groundwater sample into four distinct types i) Ca^2+^-Mg^2+^–HCO_3_^-^, ii) Ca^2+^-Mg^2+^- SO_4_^2-^, iii) Na^+^-Cl^-^, iv) Na^+^-HCO_3_^-^ (Piper, 1944; Musa et al., 2014). The predominant water samples in fallow the order of Ca^2+^-Mg^2+^-SO_4_^2-^ > Ca^2+^ -Mg^2+^–HCO_3_^-^. About 65% of water sample belongs to Ca^2+^-Mg^2+^-SO_4_^2-^ and 35% is locked to Ca^2+^-Mg^2+^–HCO_3_^-^ water types (Figure 5). This is due to rock-water interaction, reactions within unsaturated zones, ion exchange and reversed ion exchange, anthropogenic influences, and increased resident time (Nazzal et al., 2014). The most important fact is that Mg^2+^ concentration in 73% of water samples and Ca^2+^ in 24% of samples exceed the WHO drinking water quality standards. No doubt, both are essential nutrients for animals and plants but their excessive ingestion through drinking water could result in bone damage and nerve system disordering (Tiwari et al., 2017).

### 3.5 Principal component analysis

The PCA was executed to authorized the contributed factors toward the concentration, distribution, and source detection of ions in groundwater. The significant principal component number were selected depending on varimax rotation with Kaiser normalization with eigenvalues higher than 1. Four principal components (e. g. PC1, PC2, PC3, PC4) have eigenvalues greater than 1, which represent 70.179% total variance of the data set. For data interpretation, the component loading factor >0.6 was considered (Singh *et al.*, 2017). The PC1 represented 35.201% of total variance and high positive loading value of EC (0.826), TDS (0.799), Mg^2+^ (0.785), PO_4_^3-^ (0.710), SO_4_^2-^ (0.667) and Cl^−^ (0.744). While pH (−0.701) shows a high negative loading value (Table 2). The high loading factor in PC1 represented that anion and cation in groundwater arise from natural and anthropogenic sources. The weathering and leaching of host rocks is the main natural source, while anthropogenic sources are municipal solid waste dumping, sewage discharge, and fertilizer application (Yadav et al., 2018). The high EC and TDS concentration arises from elevated dissolved minerals in water (Singh *et al.*, 2017). The presence of Mg^2+^ and SO_4_^2+^ in PC1 suggests that agricultural activities are another contributing mechanism.

**Table 2:** Rotated factor loading matrix, eigenvalues, % variance and cumulative variance values.

Parameter	PC 1	PC 2	PC 3	PC 4
pH	**-0.701**	0.137	0.213	-0.079
Turbidity	0.258	0.080	**-0.783**	0.140
EC	**0.826**	0.499	-0.029	-0.091
TDS	**0.799**	0.511	-0.021	-0.159
Na^+^	0.238	0.331	0.424	0.586
K^+^	0.452	**-0.676**	0.041	-0.255
Ca^2+^	0.454	-0.074	0.362	**-0.633**
Mg^2+^	**0.785**	-0.381	0.142	0.186
PO_4_^3-^	**0.710**	-0.436	0.094	0.252
SO_4_^2-^	**0.667**	-0.576	0.048	0.173
NO_3_^-^	0.326	0.430	0.067	0.029
HCO_3_^-^	0.078	0.409	0.389	0.075
Cl^-^	**0.744**	0.452	-0.207	-0.109
Eigenvalue	4.576	2.331	1.201	1.014
% Variance	35.201	17.934	9.240	7.803
Cumulative %	35.201	53.135	62.375	70.179
Extraction Method: Principal Component Analysis.				

While the presence of Cl^−^ and PO4^3-^ indicates the influence of municipal wastes and onsite sanitation on groundwater (Mohapatra et al., 2011). This is also suggest that waste dumping and agricultural activities occur in the area for a long period (Satyaji *et al.*, 2010). The PC2 accounts for 17.934% of total variance with high negative loading of K^+^ (−0.676). While PC3 accounts for 9.24% of total variance with high negative loading of turbidity (−0.783). Both K^+^ and turbidity have a negative correlation with pH (not shown in the table). The PC4 account for 7.803% of total variance with high negative loading of Ca^2+^ (−0.633). The high concentration of Ca^2+^ and Mg^2+^ was found along the study area but doesn't originate from the same source of minerals.

## 4 Conclusions

Groundwater is an important resource widely used for drinking and irrigational purposes in Bangladesh. The groundwater is slightly alkaline in the study area. The water is highly turbid and 68% of the sample exceeds WHO drinking water quality standards. The EC value ranged from 570 to 2100 μS cm^−1^ with a mean value of 891.80 (±242.45) μS cm^−1^. The spatial distribution showed that the concentration of the majority of ions gradually increases from north to south (Bay of Bengal) along the study area. Two distinct hydrogeochemical facies Ca^2+^-Mg^2+^- SO_4_^2−^ and Ca^2+^-Mg^2+^–HCO_3_^-^ was predominant in the study area. The PCA results shows that weathering and leaching of host rocks is the main natural source for releasing ions in groundwater, which is supported by the Gibbs plot. The Gibbs plot suggests that dissolution and weathering of minerals along with the ion-exchange process is the main force for controlling groundwater chemistry, while precipitation and evaporation don't have a significant influence. It is also observed from weathering and dissolution diagram that carbonate weathering is the predominant process of releasing those ions. It is also suggestive that weathering of gypsum, calcite, dolomite, and other carbonate-bearing minerals is active along the study area. Hence this is the fast baseline data in this area and may consider for further planning while using this water for drinking purposes. However, further study is recommended to provide a more scientific basis and identification of trace metallic pollutants in the study area.

## Declarations

### Author contribution statement

Tusar Kumar Das: Conceived and designed the experiments; Analyzed and interpreted the data; Contributed reagents, materials, analysis tools or data; Wrote the paper.

Alamin Hossen; Shakir Ahmed: Performed the experiments; Wrote the paper.

Mohammad Mahfuzur Rahman; Md. Hasibur Rahaman: Contributed reagents, materials, analysis tools or data; Wrote the paper.

### Funding statement

This research did not receive any specific grant from funding agencies in the public, commercial, or not-for-profit sectors.

### Data availability statement

Data will be made available on request.

### Declaration of interest's statement

The authors declare no conflict of interest.

### Additional information

No additional information is available for this paper.
